# Midterm results of posterior arthroscopic ankle fusion

**DOI:** 10.1007/s00167-015-3975-z

**Published:** 2016-01-12

**Authors:** Peter A. J. de Leeuw, Roel P. M. Hendrickx, C. Niek van Dijk, Sjoerd S. Stufkens, Gino M. M. J. Kerkhoffs

**Affiliations:** Department of Orthopaedic Surgery, Orthopaedic Research Center Amsterdam, Academic Medical Center, PO Box 22660, 1100 DD Amsterdam, The Netherlands; Department of Orthopaedic Surgery, Zuyderland, Sittard, The Netherlands; Academic Center for Evidence Based Sports Medicine (ACES), Amsterdam, The Netherlands; Amsterdam Collaboration for Health and Safety in Sports (ACHSS), Amsterdam, The Netherlands

**Keywords:** Ankle, Fusion, Arthroscopy, Osteoarthritis, Hindfoot endoscopy

## Abstract

**Purpose:**

The presented study was performed to evaluate the midterm clinical and radiological results and complication rates of the first 40 patients with an ankle fusion through a posterior arthroscopic approach.

**Methods:**

Forty consecutive patients with end-stage post-traumatic ankle osteoarthritis were treated with posterior arthroscopic ankle fusion. All patients were assessed clinically as well as radiologically with a minimum follow-up of 2 years. The Foot and Ankle Ability Measure (FAAM) and Foot Function Index (FFI) were used to assess clinical improvement.

**Results:**

Clinical fusion was achieved in 40 patients within 3 months (100 %), and radiological fusion was achieved in 40 patients at 12 months. Two screw mal-placements occurred. Both complications were solved following revision surgery. A significant improvement was noted for both the FAAM [median 38 (range 17–56) to 63 (range 9–84)] and FFI scores [median 66 (range 31–89) to 32 (range 11–98)] for all 40 patients.

**Conclusion:**

The posterior arthroscopic ankle fusion is an effective and safe treatment option for end-stage post-traumatic ankle osteoarthritis at midterm follow-up.

**Level of evidence:**

Prospective cohort study, Level IV.

## Introduction

Several methods have been described for fusion of the ankle joint. Secure fusion can be accomplished arthroscopically, by open surgery or by using a so-called mini-open technique [5, 9, 17–19]. An open procedure might allow for a better correction of mal-alignment of the hindfoot, when significant deformity is present. However, recent evidence supports the use of arthroscopic techniques for more markedly deformed ankles as well [6]. Arthroscopy assisted fusion is becoming more popular with equivalent high union rates, but lower complications, as compared to open surgery [5, 19, 21].

The posterior ankle arthroscopic technique, also known as hindfoot endoscopy, is safe and reliable [7, 10]. A posterior arthroscopic approach allows for two important biomechanical advantages: enhanced primary stability can be achieved through contour-shaped cuts, and optimal compression can be applied through parallel screw positioning [10, 13, 15, 25]. Prior to commencing the presented technique in our patients, an anatomical study was performed to evaluate its safety and efficiency [10].

The purpose of this prospective cohort study was to assess the midterm results of the first 40 ankle fusions in which the posterior ankle arthroscopic surgical technique was used [11].

## Materials and methods

Between 2010 and 2013, a consecutive series of 40 ankles in 40 patients were treated with posterior arthroscopic ankle fusion. All surgeries were performed by a single surgeon (GK). The inclusion criteria were patients with end-stage post-traumatic ankle osteoarthritis refractory to conservative treatment options. Exclusion criteria were revision fusion surgery, failed ankle prosthesis or double fusions.

**Fig. 1:**
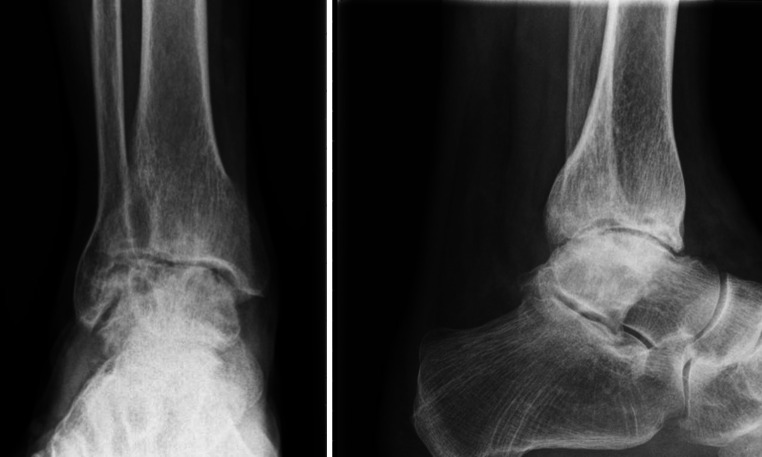
Weight-bearing radiographs in the anterior-to-posterior and lateral direction with end-stage post-traumatic ankle osteoarthritis

Preoperative data consisted of a common clinical evaluation (history and physical examination) and plain radiographs in orthogonal planes (Fig. 1). In addition validated subjective outcome instruments, such as the Foot and Ankle Ability Measure (FAAM) and a Foot Function Index (FFI), were obtained [2, 4, 16].

### Surgical technique

The standard two-portal technique for hindfoot arthroscopy was used [23, 24]. Routine instruments for debridement of the ankle joint consisted of a 5.5-mm platinum Dyonics Bonecutter shaver blade (Smith&Nephew^®^), small curette and a 5.0-mm chisel. In selected cases, when a “bowler-hat”-shaped talus was present an accessory anteromedial portal was used for debridement of the anterior rim of the talus [11]. The technique allows for debridement of >95 % of the ankle joint [10].

Following debridement of the ankle joint a limited trans-Achilles tendon approach was used for insertion of two parallel orientated non-cannulated 6.5-mm partially threaded cancellous compression screws. This approach was preferred over the posteromedial or lateral ankle approach due to the good working area with the ability to nicely orientate the screws. Screw insertion was performed under fluoroscopic guidance.

### Post-operative rehabilitation

Patients were kept in a non-weight-bearing cast for 6 weeks. Following clinical and radiological assessment, a weight-bearing cast (or walker) was applied for another 6 weeks. Once a solid fusion was obtained, the patient was allowed to wear regular shoes and could resume activities as tolerated.

### Clinical and radiological evaluation

The follow-up protocol demanded regular visits at 2, 6, 12 and 52 weeks post-operatively. Additionally, 2 years following surgery an evaluation of all patients included abilities to sport and physical functioning, the FAAM score and a FFI score [2, 16].

The FAAM comprises the separately scored 21-item ADL and eight-item sports subscales. Each item is scored on a five-point Likert scale anchored by 4 (no difficulty at all) and 0 (unable to do). Item score totals, which range from 0 to 84 for the ADL subscale and from 0 to 32 for the sports subscale, are transformed to percentage scores. A higher score represents a higher level of function for each subscale [16]. The FFI consists of 23 items grouped in three subscales (limitation in activity, disability and pain). Visual analogue scales are used for each item. The subscale scores are averaged together to obtain a total mean score. A lower FFI score represents a higher level of function [2].

Radiographs were obtained immediately post-operative and at the regular controls (6, 12 and at 52 weeks post-surgery). A standard anterior-to-posterior and lateral X-ray of the ankle without a cast was obtained to assess fusion. Clinical union was based on lack of ankle motion and full weight bearing without pain, whereas radiological union was defined as bridging trabeculae in two radiographic planes [8]. Institutional review board approval from the University of Amsterdam, the Netherlands, for the prospective inclusion of the patients was obtained under registration number W14_237 #14.17.0288.

### Statistical analysis

Statistical analysis was performed using SPSS 23 for Mac (SPSS Inc, Chicago, Illinois). Single *t* test for repeated measures was used. Significance levels of *p* < 0.05 were used throughout this study. Additionally, post hoc power analysis was performed.

## Results

The median follow-up on 40 patients was 42 months, with a range of 24–66 months. The study population consisted of 24 males (60 %) and 16 females (40 %). The median age was 53 years old (range 21–80). The median operation time was 93 min (range 63–121). All surgeries were performed in the hospital with a single overnight stay.

**Fig. 2:**
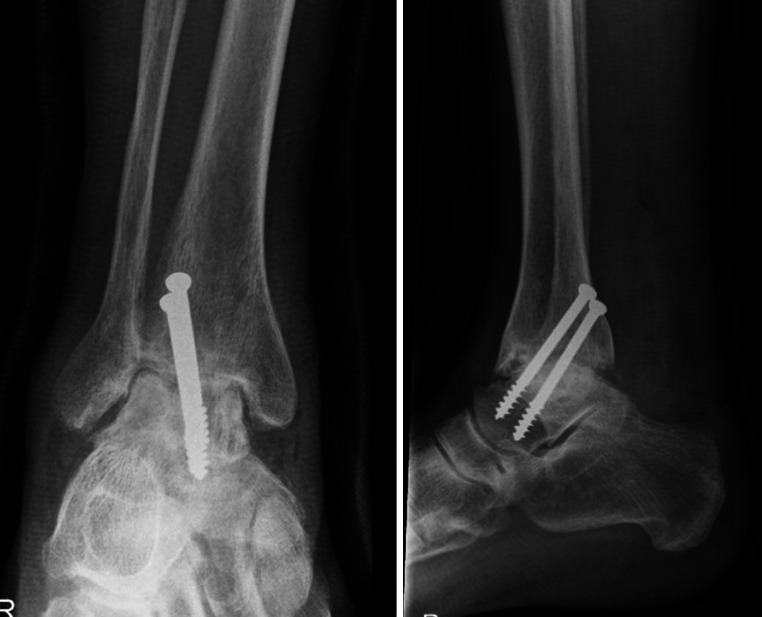
Radiographs in the anterior-to-posterior and lateral direction at 2 weeks following posterior arthroscopic ankle fusion with the two screws in an orientation from posterolateral to anteromedial over the ankle joint

**Fig. 3:**
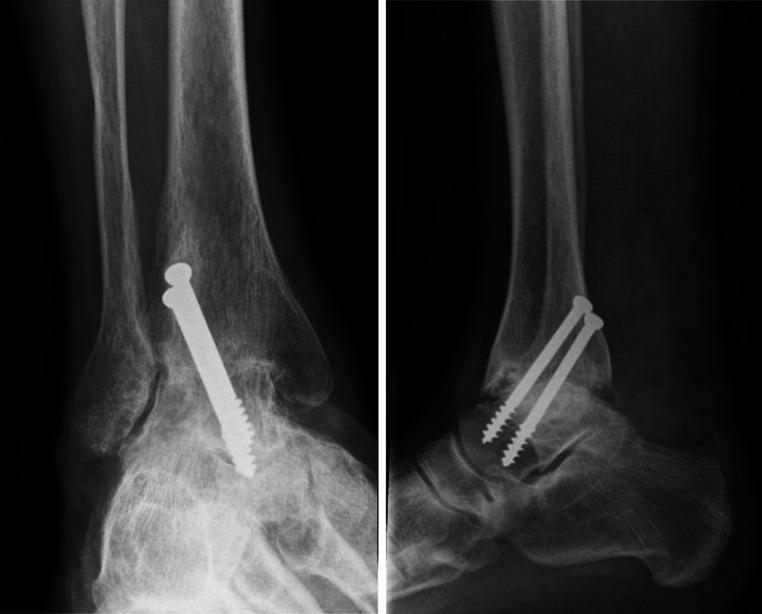
Weight-bearing radiographs in the anterior-to-posterior and lateral direction at 1-year follow-up with union in both radiographic planes

At 3 months post-operatively, 40 patients (100 %) were fused on clinical evaluation, whereas radiological union was achieved at 12 months post-operatively in all 40 patients (Figs. 2, 3). Two patients (5 %) needed secondary surgery. The reasons for secondary surgery were screw mal-placements; however, these patients both united within 3 months of the index surgery. There were no post-operative infections or transient or permanent neurovascular injuries

### Return to sports

Prior to suffering from ankle OA, the majority of the treated patients were active in some field of sports. Recreational sports (golf, swimming, hiking) were most commonly performed by 48 % of the subjects. Fourteen per cent were active in endurance sports (running, biking). Twenty per cent was performing contact sports (soccer, hockey, martial arts).

At the time our population suffered from end-stage OA of the ankle, 59 % were unable to participate in any sports activity. A substantial group of 41 % remained active, however, in recreational sports. No subject was able to perform endurance or contact sports after the ankle fusion surgery.

Two years following surgery, a shift in sportive capabilities was seen. Still 37 % was not sportively active. Fifty per cent performed recreational sports. Thirteen per cent had (actively) joined a fitness programme (Table 1).

**Table 1:** Sports behaviour

	No. sports (%)	Recreational sports (%)	Endurance sports (%)	Contact sports (%)	Fitness (%)
Prior to OA	19	48	14	19	0
End-stage OA	59	51	0	0	0
Two-year follow-up	37	50	0	0	13

### Patient-reported outcome measures

Self-reported outcome measures were taken prior and at 2 years following surgery. For this purpose the Foot and Ankle Ability Measure and FFI were used [2, 26] (Table 2).

**Table 2:** Outcomes of self-reported questionnaires

	Median	Range	*p* value
FAAM baseline	38	(17–56)	
FAAM 2 year	63	(9–84)	<0.05
FAAM sport baseline	2	(0–9)	
FAAM sport 2 year	4	(0–20)	<0.05
FAAM total baseline	42	(17–63)	
FAAM total 2 year	71	(9–96)	<0.05
FFI baseline	66	(31–89)	
FFI 2 year	32	(11–98)	<0.05

The FAAM ADL and sports subscales were scored separately. The values for ADL increased from a median of 38 (range 17–56) to 63 (range 9–84). This increase is statistically significant (*p* < 0.05), with a power of 99 %.

The FFI decreased from 66 (range 31–89) to 32 (range 11–98). This decrease is statistically significant (*p* < 0.05), also with a power of 99 %.

## Discussion

The main finding of this study is that an ankle fusion through a posterior arthroscopic approach is an effective and safe technique to treat end-stage post-traumatic ankle OA with an union rate of 100 % in the first 40 cases. Two (5 %) complications were encountered. Patient-reported outcome measures improved significantly after surgery.

Arthroscopic ankle fusion is an established treatment option for end-stage ankle OA resulting in equivalent union rate, lower complication rate and shorter hospital stay, as compared to open-ankle fusions [1, 20, 22]. The most reported arthroscopic techniques to fuse the ankle joint use anterior portals [3, 12, 27]. However, in most post-traumatic (and instability-induced) osteoarthritic ankles, the anterior part of the joint has the least remaining cartilage, the worst soft tissues after earlier surgeries and therefore presents less proper accessibility to the ankle joint in some cases and higher chance of wound problems and subsequent infections. Additionally, through an anterior approach, the posterior part of the ankle joint is less accessible. Although the most posterior part of the talar surface might not be of utmost importance to achieve a solid fusion, we feel that the posterior part of the tibial plafond do is very important in obtaining union. With the posterior approach, we are able to debride this part meticulously, and with the direction of the compression forces from the two screws, the talar surface is pooled circular into this posterior part of the distal tibial plafond, allowing early fusion over a large posterior surface area. We consider a posterior approach, therefore, a more suitable technique allowing good access, enabling more accurate compression on the joint surfaces and probably also a more proper removal of the remaining cartilage. Recently, Malekpour et al. performed an anatomical study in 10 specimens to assess the effectiveness of posterior arthroscopy for debridement of the ankle joint [14]. In all specimens complete debridement of the tibia plafond was noted. On the talar side about 80 % could be addressed. The anterior one-third of the talar dome was hard to reach. Prior to the current clinical study, we conducted an anatomical study as well. With the described technique, we were able to debride 96 % of the articular surface of the entire ankle joint [10].

The overall non-union rates after anterior arthroscopic assisted ankle fusions are 8.6 % [1]. The union rates in the presented study compare favourably to these. To achieve a bony fusion, both an adequate debridement and compression is needed. The presented technique addresses both of these principles, and possibly therefore, the presented union rates were achieved. However, we are the first to realize that this percentage will be lower with an increase in the patient number.

In two cases an early revision surgery was indicated; the screw mal-placements were in the first 10 operated patients. These complications might be marked as part of the learning curve, and in the first operated patients, screw length was maximized up to the subcortical talocalcaneal joint border to assure for optimal compression. In the consecutive patients, diminished screw lengths were used which apparently did not result in diminished union rates.

The Foot and Ankle Ability Measure (FAAM) showed significant increase for ADL and sportive activity in our patients after 2-year follow-up. However, only improvement in the ADL subscale can be considered clinical relevant according to the instructions for use of the FAAM scoring system [16]. Following fusion a significant improvement in the Foot Function Index (FFI) was noted. These findings are similar to the current literature [22].

## Conclusion

This midterm follow-up study shows that posterior arthroscopic ankle fusion is a promising surgical technique to perform an ankle fusion in patients with post-traumatic osteoarthritis.
